# Intrathecal morphine delivery at prepontine cistern to control refractory cancer-related pain: a case report of extensive metastatic and refractory cancer pain

**DOI:** 10.1186/s12871-024-02426-8

**Published:** 2024-02-26

**Authors:** Qing Li, Yan-ling Long, Yun-wu He, Hui Long, Zhen-ping Xiao, Yong-lin Li, Wu-zhou Yang, Li-ping Jiang, Wei Gao, Cong Zou

**Affiliations:** Department of Pain and Rehabilitation, The Second Affiliated Hospital, University of South China, Hengyang, Hunan 421001 China

**Keywords:** Extensive metastatic and refractory cancer pain, Prepontine cistern, Intrathecal delivery, Subarachnoid, Opioid receptors, Numeric rating scale (NRS), Morphine, Adverse effects

## Abstract

**Background:**

Extensive metastatic and refractory cancer pain is common, and exhibits a dissatisfactory response to the conventional intrathecal infusion of opioid analgesics.

**Case Presentation:**

The present study reports a case of an extensive metastatic esophageal cancer patient with severe intractable pain, who underwent translumbar subarachnoid puncture with intrathecal catheterization to the prepontine cistern. After continuous infusion of low-dose morphine, the pain was well-controlled with a decrease in the numeric rating scale (NRS) of pain score from 9 to 0, and the few adverse reactions to the treatment disappeared at a low dose of morphine.

**Conclusions:**

The patient achieved a good quality of life during the one-month follow-up period.

## Background

Refractory cancer pain refers to the moderate or severe pain caused by the tumor itself or tumor treatment-related factors, and an unsatisfactory effect and/or intolerable adverse reaction occurs after 1–2 weeks of standardized drug treatment [1]. For patients with widely metastasized tumors, the pain is mostly refractory, which not only brings a huge economic burden to patients, but also has a huge impact on the physical and mental health of patients. Thus, refractory pain has become a thorny problem for both doctors and patients.

Intrathecal analgesia delivery, which was first reported in 1978 by Wang JK [2], has been increasingly used to treat refractory pain in clinical practice. The intrathecal catheter tip is generally placed by subarachnoid puncture near the spinal segment where the pain is the greatest, and opioids are continuously injected to control the pain. Compared to the traditional oral route, intrathecal delivery produces a better analgesic effect, with smaller doses and fewer adverse reactions [3, 4]. A number of intractable cancer pain patients have achieve relief with conventional intrathecal therapy. However, most patients with primary or metastatic cancer in the middle thoracic vertebrae or above do not achieve sufficient pain relief [5, 6]. The placement of the catheter at a higher level has advantages in reducing the analgesic dose. For example, the intrathecal delivery drug at the C1 level reduced the morphine from 1,000 mg to 300 mg [7], and significantly reduced the adverse effects related to analgesics. Recently, the prepontine cisternal routine has received increasing attention for intrathecal drug delivery to treat high cervical pain, due to the easy accessibility of the drug to nerves that innervate the pain area [8–10].

The present study reports a case of an extensive metastatic esophageal cancer patient with severe intractable pain, who presented with significant relief of pain, and a decrease in daily morphine dose after the intrathecal targeted delivery of morphine, through the placement of the catheter tip near the prepotine cistern.

## Case presentation

**Fig. 1:**
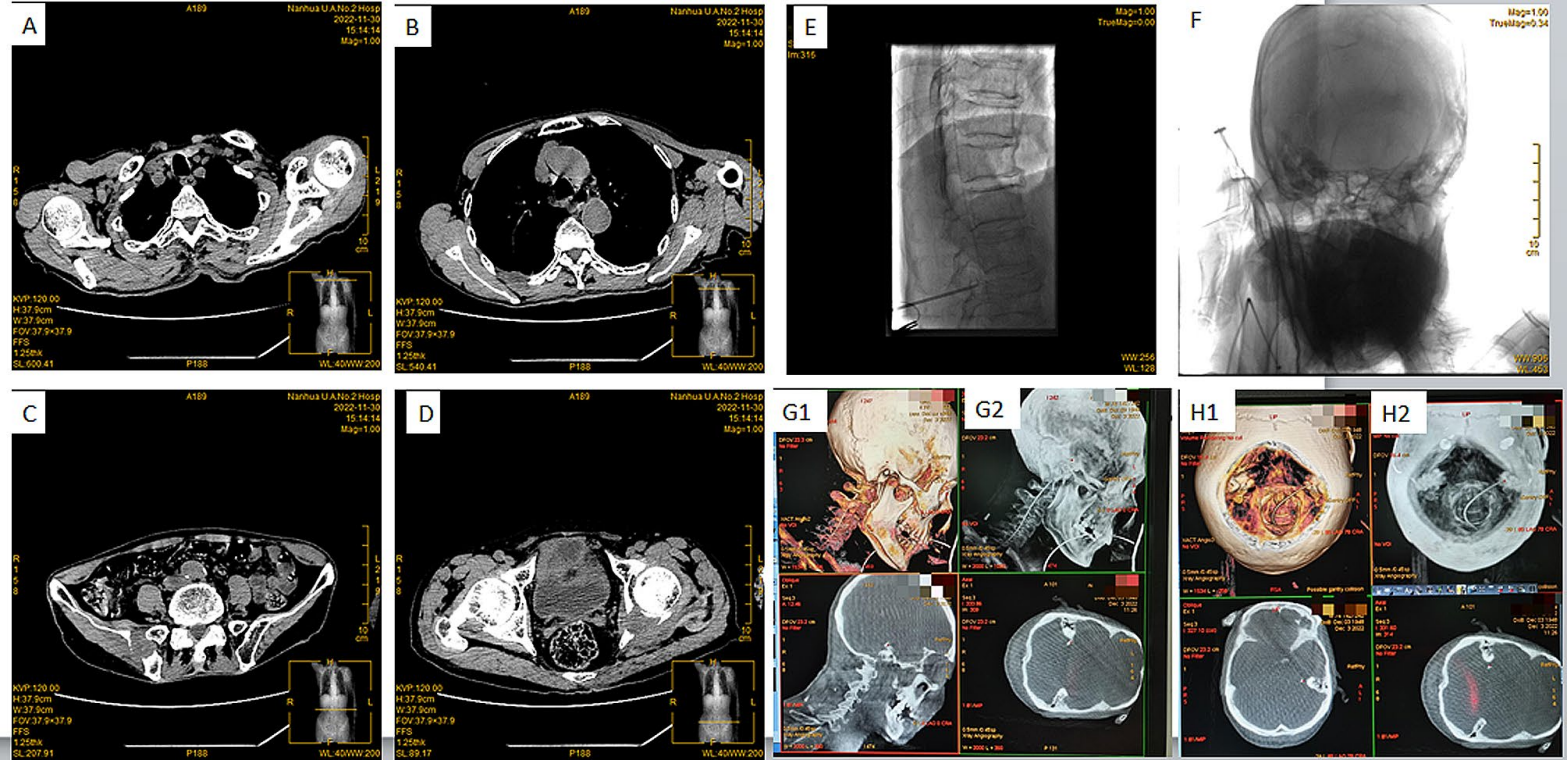
**(A-D)** The computed tomography images of the patient shows **(A)** the thickening of the esophageal wall (white arrow), **(B)** the rib bone lesion (blue circle), **(C)** the lesion on the right iliac crest (yellow circle), and **(D)** the lesion on the left ischium (red circle). The digital subtraction angiography shows **(E)** the catheter in the spinal canal, and **(F)** the catheter tip near the prepontine cistern. The 3D construction images shows the catheter **(G1-2)** and **(H1-2)** reconstruction of the skull base to the real location of the catheter

The patient was in the 70s, and was diagnosed with advanced esophagus cancer with lesions to the right rib, left ischium, and right iliac crest by computed tomography (CT) (Fig. 1A and D). The patient experienced constant severe pain around the region of the upper chest, left ischium, and right iliac crest. The pain made it difficult for the patient to move, sleep, and eat due to the narrowed esophagus. This deteriorated the pathological condition of the patient, and made the pain refractory to oxycodone hydrochloride (sustained release tablet, 120 mg, *q12h*). The numeric rating scale (NRS) of pain score was 6 at rest, and 8–9 in action. The patient and family members were informed of different options and potential risks, but refused other options of radiotherapy and chemotherapy. They agreed with the intrathecal perfusion for good life quality, and signed the consent form. Due to economic problems, the family members refused other neurological examinations, such as imaging. The patient was treated with cefuroine due to the pulmonary infection before the intrathecal perfusion, and was treated with cefoperazone after the intrathecal perfusion due to fever.

Considering the wide range of pain of the patient, and the uncertain efficacy of the intrathecal spinal infusion, the catheter was implanted in the prepontine cistern for the intrathecal delivery of morphine. The patient was placed in the left lateral decubitus position, and the L2/3 spinous space was located under the guidance with digital subtraction angiography (DSA). After routine disinfection and placement of the sterile surgical towel, lumbar puncture was performed *via* the gap between L2/3 using a Tuohy needle, until the needle tip reached the subarachnoid space, and the cerebrospinal fluid reflux became visible. Then, under the continuous guidance of DSA, the intrathecal catheter was slowly placed into the prepontine cistern through the lumbar, thoracic and cervical spinal subarachnoid space, and finally, through the foramen magnum. During the placement of the catheter, the cerebrospinal fluid outflow remained unobstructed. The position of the catheter on the clivus was confirmed through lateral radiographs, and the position of the catheter tip in the prepontine cistern was confirmed through 3D-CT scans (Fig. 1E and F, 1G1, 1G2, 1H1 and 1H2). Then, a subcutaneous tunnel was established at puncture point L2/3 and the right mid-abdomen to bury the catheter. Subsequently, a morphine infusion pot connecting catheter was inserted into the subcutaneous tissue at the right mid-abdomen, and a butterfly needle was used to connect the drug infusion pot to the external analgesic pump. The patient experienced no discomfort during the whole procedure.

**Fig. 2:**
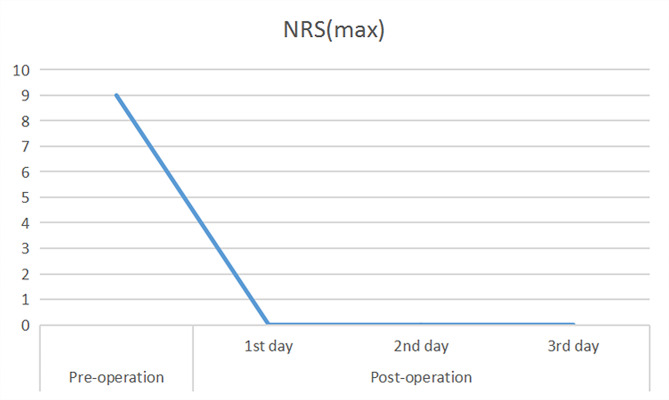
Comparison of NRS scores of the patient before and after the intrathecal infusion of morphine

Through the equivalent conversion relationship between oxycodone and morphine, the morphine concentration in the analgesic pump was set to 0.033 mg/ml, with an initial rate of 0.3 ml/h. The pain was significantly relieved after the operation, and the NRS score was 0. However, the patient presented with significant vomiting and urinary retention on the second day after surgery. Thus, the morphine concentration was adjusted to 0.0165 mg/ml with a pumping speed of 0.2 ml/h. As a result, the vomiting and uroschesis were relieved, the NRS of pain score remained 0, and there was no breakthrough pain episode (Fig. 2; Table 1). In addition, the patient smoothly recovered, and was allowed to eat porridge and noodles. Both the patient and the family were very satisfied with the results of the intrathecal analgesic therapy. After three days of observation, the patient was stable, and was discharged. The NRS score remained 0 until the patient died due to the primary disease (approximately three months of follow-up from the placement of the delivery pump).

**Table 1:** Effects of the intrathecal delivery of morphine in patients

	Pre-operation	1st day	2nd day	3rd day
NRS (max)	9	0	0	0
Eating disorder	√	/	/	/
Emesis	/	√	√	/
Uroschesis	/	√	√	/

NRS, numeric rating scale

## Discussion and conclusions

Since the first report [1], intrathecal analgesia has been increasingly used in clinical practice to control refractory pain, which remains as a difficulty for patients, families, and the society. The direct intrathecal infusion of analgesics can induce the drug to bypass the blood-brain barrier (BBB), reach the central nervous system, and immediately bind to the receptors [3, 4]. Therefore, the intrathecal injection of morphine and hydromorphone has been widely used for the treatment of pain in advanced cancer below the upper thoracic segment, and this has achieved a relatively ideal analgesic effect [11].

Previous studies have indicated that the location of the catheter for delivering the analgesia may have an influence on the analgesic effect. Sun [8] reported that, compared to the injection of morphine in the midthoracic spinal cord segment, the CT-guided percutaneous puncture injection of morphine into the cisterna magna led to a better analgesic effect for patients with refractory pain above the middle thoracic vertebrae level. This led to the decrease in dose of morphine to 24–42%, which is helpful for preventing adverse effects. Furthermore, Zou [9] and Zhou [12] reported that placing the catheter tip near the prepontine cistern through lumbar puncture can well-control the pain of patients with terminal craniofacial cancer. Consistently, the patient in the present study reached a satisfactory analgesic effect, even with a low dose of morphine, by placing the catheter tip in the prepontine cistern. The prepontine cistern is the subarachnoid space located dorsally to the clivus and ventrally to the pons, and this communicates with the subarachnoid space of the spinal cord through the foramen magnum. Due to its anatomical property, the prepontine cistern is accessible for placement of the catheter tip from the subarachnoid space of the lumbosacral segment. After the prepontine cistern delivery of morphine, the patient in the present study presented with vomiting and urinary retention which are common adverse effects of morphine treatment. These were significantly relieved after decreasing the dose of morphine. Compared to the oral route, the dose of morphine was significantly reduced after surgery, and the analgesic effect remained excellent.

Cancer pain arising from disease pathology and/or cancer treatment is a complex condition driven by inflammatory, neuropathic, and cancer-specific mechanisms. The peripheral cross-talk among tumor cells, non-neuronal cells, and neurons is a key process for the induction and maintenance of cancer pain states [13]. Although the etiology of cancer pain remains unclear, animal models of cancer pain have allowed investigators to unravel some of the cancer-induced neuropathologic processes that occur in the region of tumor growth, and in the dorsal horn of the spinal cord. Within the cancer microenvironment, cancer and immune cells produce and secrete mediators that activate and sensitize primary afferent nociceptors through a variety of receptors on peripheral nociceptive nerve terminals, in order to promote abnormal discharge and hyperexcitability [14]. The placement of the catheter for drug delivery *via* the pump plays an analgesic effect through the direct continuous diffusion of the drug to nerves that innervate the pain region, resulting in satisfactory effects for pain relief.

The satisfactory analgesic effects of the prepontine cistern placement of the catheter may be correlated to the distribution of opioid receptors in the central nervous system. Previous studies [10, 15] have indicated that opioid receptors are mainly distributed in the brain, especially in the periaqueductal gray matter, amygdala, midline thalamic nuclei, and spinomesencephalic tract, but are less distributed in the spinal subarachnoid space.

There are several technical notes for placing the catheter tip to the prepontine cistern. The preoperative imaging examination of the patient’s head and spine should be performed to exclude serious intraspinal occupying lesions, and ensure a smooth operation. Furthermore, when placing the catheter, the operation should be gentle and slow under the guidance of the DSA. Moreover, when there is any resistance, the movement should be stopped to check the catheter position, and carefully adjust the catheter tip. Finally, 3D CT scans should be performed to confirm the position of the catheter tip on the prepontine cistern [16]. However, these neurological examinations were declined by the patient and family members due to economic problems.

In summary, the present study indicates that intrathecal morphine delivery at the prepontine cistern is an effective therapeutic approach for pain control in patients who suffer from extensive metastatic and refractory cancer pain. This application can reduce the dose of opioid required to reach a satisfactory analgesic effect, and has the least side effects. However, it is necessary to conduct prospective studies with a larger sample size in the future to further confirm its clinical safety/efficiency and related mechanisms.

## Data Availability

The datasets used and/or analyzed in the study are available from the corresponding author upon reasonable request.

## Abbreviations

NRS: numeric rating scale
CT: computed tomography
DSA: digital subtraction angiography
BBB: bypass the blood-brain barrier
